# Conservative treatment of juvenile with Chiari I malformation, syringomyelia and scoliosis. Two case reports

**DOI:** 10.1186/1748-7161-8-S1-O52

**Published:** 2013-06-03

**Authors:** M Rigo, B Janssen, R Campo, L Tremonti

**Affiliations:** 1 Institut Elena Salvá, Barcelona, Spain; 2 ScoliosisRehab, Stevens-Point, Wisconsin, USA

## Background

Scoliosis improvement after surgical treatment of Chiari I and syrinx has been reported [1]. Incidence of scoliosis progression after decompression surgery has been reported as high as 48%. The conservative treatment with brace in these patients is not effective and scoliosis is typically progressive [2]. Spontaneous resolution of CT syrinx and Chiari I in paediatric population is uncommon. We have previously published a unique case report of an 8-year-old girl, showing resolution of syrinx and Chiari I, as well as scoliosis reduction of scoliosis during brace treatment [3]. We present results after longer follow up, together with a new case of good response to bracing, in a girl showing scoliosis progression after neurosurgical treatment.

## Case Presentation

### First case presentation

A 7-year-old girl who showed scoliosis progression from 44º to 55º six months after neurosurgical decompression to treat Chiari I (10-11 mm tonsillar ectopia) associated with C5-T11 syrinx was subsequently recommend going under scoliosis surgery (rejected). Eight months following neurosurgery, patient began full-time treatment with a Chêneau type brace (RSC). She started later a program of specific exercises based on Schroth-Barcelona (BSPTS). At 12 years of age (5 years follow-up) she shows a 6º main thoracic curve in her 4th brace, although still at Risser 0. Formetric reports a totally regressed back asymmetry and physiological sagittal profile. The patient is asymptomatic and continues full time bracing and exercises.

### Second case presentation

A 13-year-old girl started full time bracing when she was 8 years old, after showing progression from 36º to 47º in five months in her right thoracic scoliosis associated with symptomatic Chiari I and C4-T9-10 syrinx. She showed spontaneous resolution of Chiari I and almost resolution of syrinx with no recurrence, and good response to bracing. At five years follow-up she continues partial-time bracing (16 H) and BSPTS exercises, asymptomatic, with a main thoracic curve of 17º (combined with 17º functional left lumbar), one-year post-menarche and Risser 3. Back asymmetry is totally regressed and sagittal profile physiologic.

## Conclusion

Conservative treatment should be considered in patients with Chiari I/syrinx associated to progressive scoliosis, prior to or post-neurosurgical intervention, and prior to scoliosis surgery.
